# Insights into Calcium Phosphate Formation Induced by the Dissolution of 45S5 Bioactive Glass

**DOI:** 10.1021/acsbiomaterials.4c01680

**Published:** 2025-01-21

**Authors:** Elkin Lopez-Fontal, Stéphane Gin

**Affiliations:** † The Cavendish Laboratory, University of Cambridge, Cambridge CB3 0HE, U.K.; ‡ CEA, DES, ISEC, DPME, SEME, University of Montpellier, Marcoule, Bagnols-sur-Cèze F-30207, France

**Keywords:** calcium phosphate (CaP), bioglass (BG) dissolution, electron microscopy (EM), FIB cross sections, nanocrystalline apatitic structure, ToF-SIMS analysis

## Abstract

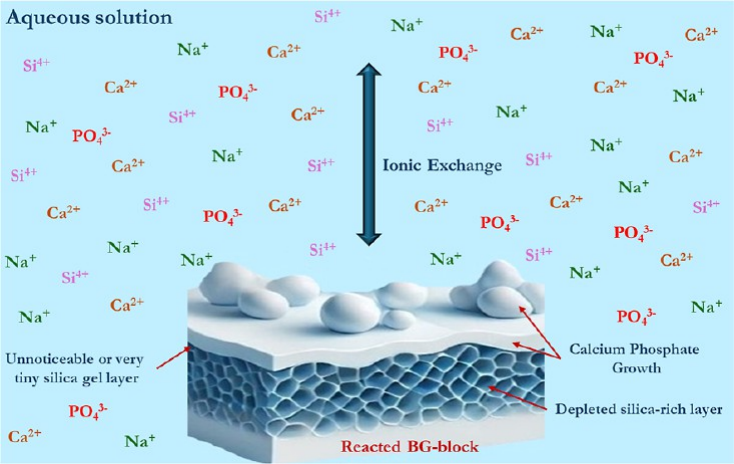

Although models have been proposed to explain the mechanisms of bioglass (BG) dissolution and subsequent calcium phosphate (CaP) mineralization, open questions remain. The processes in which phase transition occurs in aqueous solutions and their dynamics remain underexplored partly because traditional instruments/techniques do not allow for direct observations at the adequate time and length scales at which such phase transformations occur. For instance, given the crucial role of the silica gel in CaP formation during BG dissolution, uncertainty exists about how such a silica gel forms on the BG surface. In the case of CaP formation driven by BG dissolution, questions can also be added, i.e., how CaP develops into an apatitic-like structure, how many transient phases there are, and, in general, phenomena occurring in the solid–liquid interface during BG dissolution. Several approaches were taken to study CaP mineralization driven by BG dissolution, mainly examining the solid–liquid interface and the BG after-reaction surface. This paper focuses on gaining insight into silica gel formation on the BG’s surface during dissolution. Electron microscopy techniques were used, including scanning electron microscopy and focused ion beam cross sections. Other analysis techniques, such as time-of-flight secondary ion mass spectrometry, were utilized. Cross sections of reacted BG-blocks gave essential insights into the BG dissolution, particularly its strong dependency on experimental conditions, and tentative evidence has shown that soluble silica from BG dissolution may not reprecipitate/repolymerize on BG blocks’ surface; thus, we wonder where it precipitates. Additionally, complementary analysis techniques determined that CaP, during BG dissolution, transitions from amorphous calcium phosphate to a calcium-deficient nanocrystalline apatitic structure with minimal contents of Si^4+^ and Na^+^ ions that may be molecularly part of CaP. The Hench model has been the core guide for BG dissolution and subsequent CaP formation for many years. However, this study shows tentative evidence that contributes to and somewhat differs from it.

## Introduction

Self-assembly, molecular assemblies, and materials formation are intrinsically related because they characterize the generation of a new phase.^1^ Highly ordered molecular assemblies are attractive at the scientific and industrial levels as they present unique properties that could be used to engineer macro-scale functional and technologically relevant new materials from nanoscale components.^1−3^ Living organisms rely on self-assembly processes to integrate organic macromolecules and inorganic minerals to form structures like shells, bones, and teeth, with various mechanical and structural properties.^4,5^ By combining these materials, Nature has designed sophisticated biocomposites with particular and well-defined mechanical properties.^6,7^

A fundamental understanding of the mechanisms and chemical interactions involved during materials formation is vital to establishing the relationship between the function and structure, thereby improving their functionality, manufacturing cost, degradability, and design. Synthetic approaches to replicating natural constructs do not comprise the level of control, complexity, and sophistication observed in Nature. How/why particular phases and morphologies occur during, for instance, crystal formation and growth is still an open question that is being addressed using different models.^8^ A more challenging process, which is solution-mediated, is material formation when a solid phase actively intervenes. Highly complex chemical transformations are triggered by solid–liquid interfacial interactions, where ion diffusion, hydration, condensation, and solid-phase dissolution (hydrolysis) occur for the seminal event of material phase formation. Consequently, the solid–liquid interfacial chemistry that steers material growth becomes relevant, particularly calcium phosphate formation driven by a bioglass dissolution in an aqueous environment.^9^

When a BG-block reacts with an aqueous solution, several events may simultaneously occur, although a given mechanism can control the rate of the whole reaction depending on the alteration conditions (pH, temperature, and fluid composition) or the reaction progress. These parallel occurring events include (i) hydration, described as water entering the glass; (ii) ion exchange, in which metal ions, alkali and alkaline earth from the glass are replaced by hydronium ions (protonation) from the water or other ions available in solution;^10−15^ (iii) hydrolysis/condensation, based on aqueous species nucleophilic attack of the silicate glass, resulting in the formation of silanol groups on the BG surface (silica gel layer) or silicic acid that moves directly to the solution;^11−14,16^ and (iv) calcium phosphate (CaP) formation either homogeneously or heterogeneously onto the glass surface, which is promoted by the silica gel layer formed on the bioglass (BG) coupon’s surface. However, the characteristics and properties in the native liquid environment of this silica gel layer and the solid (BG)–liquid (aqueous solution) interface are mainly undetermined.

BG alteration in an electrolyte solution like nucleation, critical nucleus formation, nascent particles, heterogeneous/homogeneous material formation, attachment/detachment of particles forming a cluster, phases transition, hydrolysis/condensation, etc., occurs at tiny scales, typically nanometer length sizes. Additionally, the formation of some materials can be speedy, requiring a more rapid image acquisition. In situ microscopy techniques like confocal,^17^ scanning electron (SEM),^18^ X-ray photoelectron spectroscopy (XPS),^19^ and other techniques^20,21^ lack the spatial resolution to follow such events; hence, researchers are considering alternatives. Transmission electron microscopy (TEM) shows advancements that can be vital to tackle phenomena where spatial and temporal resolution need resolving. With a much higher readout frequency, direct detection devices^22^ have revolutionized the achievable temporal resolution of this imaging-based technique.^23^

BG are outstanding materials that can restore damaged tissues, thus boosting the quality of life and facilitating a more prolonged and healthy life expectancy. BG discovery is considered a significant breakthrough in health care, which some researchers believe is still to fulfill its potential. Bioactive glasses^15^ were initially designed to form an interfacial bonding between an implant and a host tissue, eventually enabling full integration between the body and implant.^24^ Consequently, the formation of calcium phosphate during BG dissolution is essential to integrating and assimilating with the living tissue, which determines BG functionality and bioactivity. BG studies have given relevant insight into its structural analysis,^25−27^ indirect evidence of its dissolution mechanisms,^14,16,28,29^ indirect evidence of the critical connection between silica gel and CaP formation during BG dissolution,^30−32^ surface chemistry of BG during dissolution,^33−35^ interfacial dynamics of silicate glasses in aqueous environments,^36−39^ atomistic simulations of the solid–liquid interface dynamics,^40−46^ and BG application in tissue engineering.^47,48^ However, the solid–liquid interface during BG dissolution still needs more examination and scrutiny at the appropriate time and length scales to enable resolving, for instance, dissolution processes where crystallization occurs.^49−55^ Some of the challenging topics of current and future BG research, i.e., controlled release of therapeutic ions or biomolecules, reliable BG coatings, and tunable mechanical properties, may be better tackled by improving our knowledge of BG dissolution.

This paper shows that the aqueous species can penetrate several micrometers into a BG-block surface after 1 h of immersion. The 45S5 BG open network^25,56^ and the aqueous species interaction with the glass network-modifying ions enable rapid protonation of their nonbridging oxygens (NBOs). Hence, water infiltration causes the removal of monovalent and divalent metal ions from the glass, resulting in hydrous molecules reaching (hydration) and dissociating (hydrolysis) below the BG-block surface on the newly “internal” sites created.^13,57^ Silica gel, a product of BG dissolution formed by hydrolysis/condensation, induces apatite formation^30^ on BG-blocks’ surface. Therefore, BG’ bioactivity is directly related to and dependent on the silica-matrix/surface dissolution and its activation energy,^58^ which is intrinsically associated with its composition.

This study presents work performed using BG-blocks (coupons). Part of the paper is dedicated to reporting the results and discussing the focused ion beam (FIB) cross sections of BG-blocks reacted with two phosphate-containing solutions at 37 °C as well as the findings concerning the time-of-flight secondary ion mass spectrometry (ToF-SIMS) analysis performed on this material system (BG-blocks). The last part of the paper discusses a somewhat underexplored point related to silica repolymerization on the BG-block surface.

## Materials and Methods

### 45S5 Bioglass

#### BG Composition

**Figure 1:**
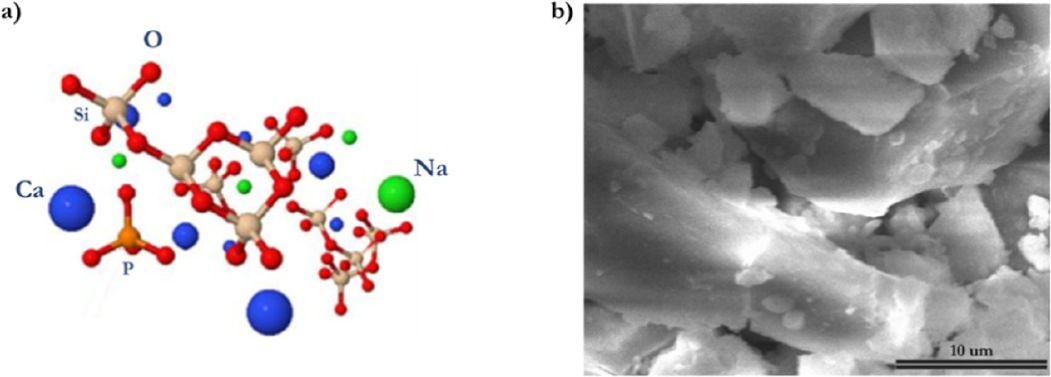
(a) Molecular structure of BG 45S5. (b) SEM micrograph of BG particles (Novamin).

The bioactive glass used in this study corresponds to 45S5 bioglass (Novamin, provided by GSK), a powdered material with a particle size *D*_50_ of ∼16 to 18 μm. Two separate phases form its molecular structure: silica and phosphate.^56^ Novamin is a silica-based modified network comprising silicon tetrahedron connectivity mainly through Q_2_ and Q_3_ connections—Q_*n*_ refers to the number of bridging oxygens within the silica network.^27^ Novamin has the following composition: silicon oxide (SiO_2_, 45 wt %), calcium oxide (CaO, 24.5 wt %), sodium oxide (Na_2_O 24.5 wt %), and phosphorus pentoxide (P_2_O_5_, 6 wt %) (Figure 1).

#### Powdered BG Melting

Powdered BG (Novamin) was melted in an electric furnace under air using a cylindrically shaped platinum/zirconium crucible. The furnace was heated at ∼15 °C/min, and before reaching the target temperature (1350 °C),^59^ it was leveled at 1250 °C for 1 h to observe the glass fluidity and general aspect. At 1250 °C, tiny bubbles appeared on the melting glass surface that may be related to the glass viscosity at this temperature or the particle size of the initial material. The plateau was then moved to 1350 °C for one and a half hours, for which the glass exhibited good transparency. Because the BG crystallizes at >720 °C, the melted glass was quenched in the air at 620 °C, annealed for 4 h at this temperature, and then naturally cooled. The cylindrical-shaped melted BG obtained was cut into discs of ∼20 mm Ø and ∼1 to 1.5 mm thick using a diamond saw; the discs were then cut into square shapes of ∼5 × 5 mm.^60^

### BG-Block Dissolution

#### BG-Blocks FIB Cross-Sectioned

**Figure 2:**
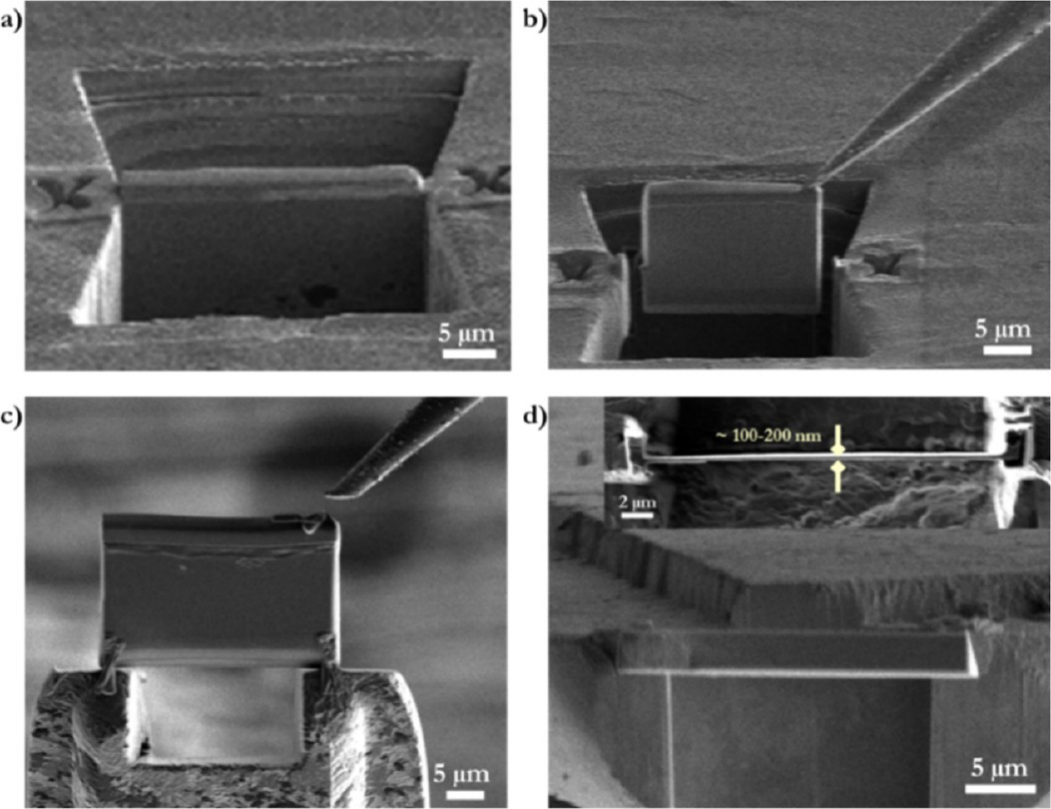
Steps to produce BG-block cross sections using FIB milling (FEI/SEM Helios). (a) Rough lamella initially produced using an automatic application (TEM-G2). (b) Cutoff lamella being lifted by a micromanipulator. (c) Lamella is placed on a copper finger in a previously prepared area. (d) Top view of a lamella ready for polishing up to TEM transparency. The inset image in (d) shows a top view of a polished lamella with the target thickness (100–200 nm) ready for TEM–EDS analysis.

BG-blocks of ∼5 × 5 mm and ∼1.5 mm thick were immersed in phosphate-containing solutions [artificial saliva (AS) and ammonium hydrogen phosphate (AmP)] at 37 °C for 1 h, after which BG-blocks were removed from the aqueous solutions and immersed in isopropanol (×3) to quench the reaction and left to dry in a desiccator.^60^ Dry BG-blocks were gold coated (∼20–50 nm) to protect the surface of reacted BG-blocks. FIB rough cross sections were prepared using an automatic application (TEM-G2), which initially produced cross sections of 20 to 30 μm in length, 10 to 15 μm wide, and 1 to 2 μm thick (Figure 2a), after which a cross-section is cut off and lifted using a micromanipulator (Figure 2b). The cross-section attached to the micromanipulator is then placed on a previously FIB-prepared area on a copper finger (Figure 2c) and polished to TEM transparency (100–200 nm) (Figure 2d and inset image).^61,62^ Polished lamellae are then EDS-TEM examined. Cross-sectioned BG-block lamellae were produced by using FIB milling (FEI/SEM Helios). EDS-TEM examinations of the produced lamellae were performed in a Thermo Scientific (FEI) Talos F200X G2 TEM—the Talos is part of the EPSRC Underpinning Multi-User Equipment Call EP/P030467/1-EDS-TEM was powered by Veloz software.

#### Time-of-Flight Secondary Ion Mass Spectrometry

A previously polished BG-block of ∼5 × 5 mm and ∼1.5 mm thick was immersed in isotopically spiked silicon-saturated aqueous solution at 37 °C for 5 h and at steady pH (∼7). The BG-block was removed from the aqueous solutions and immediately blow-dried using dry air. The reacted BG sample was then placed on the sample holder and put into a SIMS vacuum chamber. Depth profiling was performed using the sputtering mode with two primary beams to record positive ions: analysis beam Bi_1_^+^ 25 keV to 1.5pA and abrasion beam O_2_^+^ 2 keV, 600 nA. The sputtered and analyzed areas were 200 × 200 μm^2^ and 50 × 50 μm^2^, respectively. Because BG is an insulator, the profiling procedure used a low-energy neutralizing electron flux (<20 eV) after each abrasion cycle and analysis. Finally, the crater depth formed by the sputtering procedure was measured using a 3D profilometer.^39,63,64^

## Results and Discussion

### Cross Sections of BG-Blocks Reacted with Phosphate-Containing Solutions

#### BG Block Reacted with Modified AS

**Figure 3:**
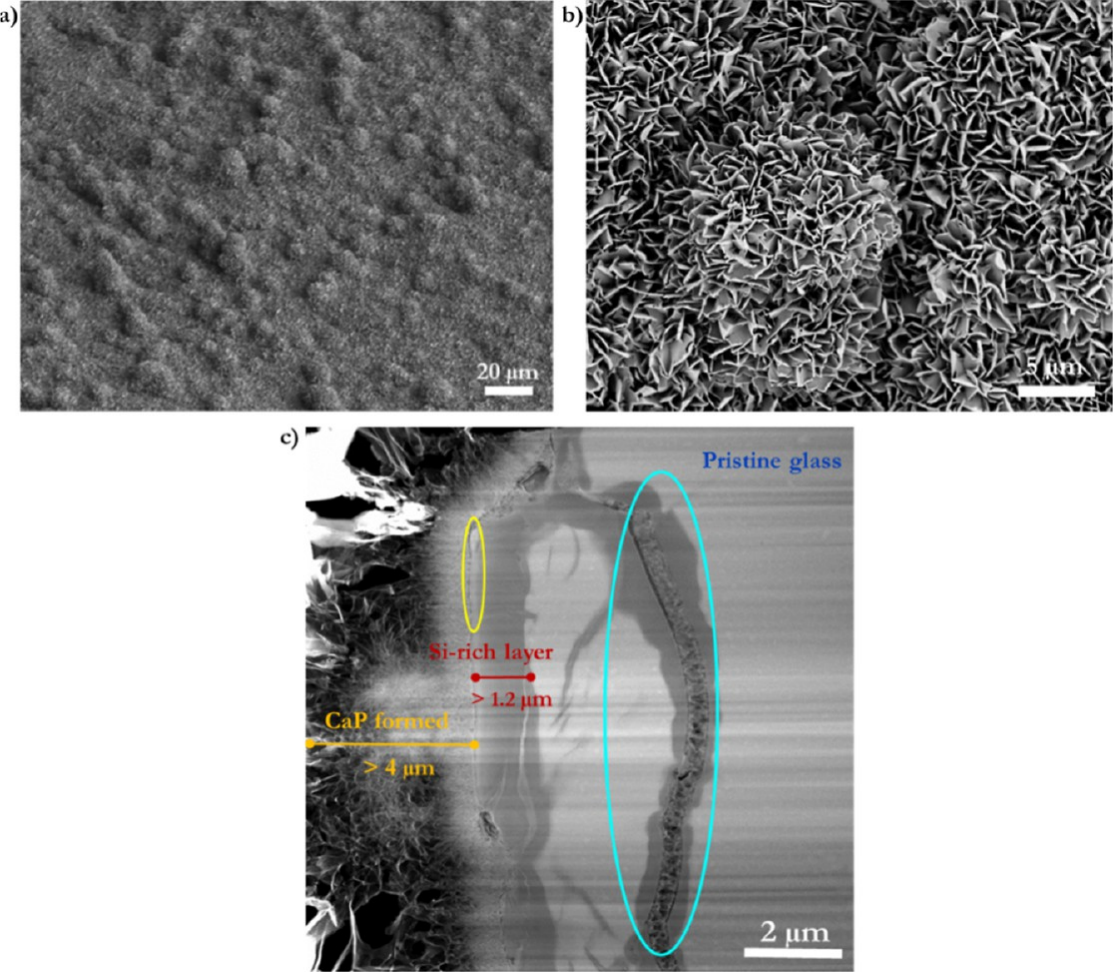
BG-block reacted with modified AS aqueous solution for 1 h at 37 °C. (a,b) SEM micrographs of the block’s surface after immersion. (c) BG-block cross-section shows some key characteristic results, such as the extent of CaP growth material (orange line) and glass modifier ion depletion (red line). Two features are highlighted: (i) a natural BG-block canal/crack in which aqueous species freely moved, showing CaP growth throughout the fissure (cyan-circled area). (ii) light-yellow circled area shows a thin layer corresponding to dense Ca^2+^ ions.

**Figure 4:**
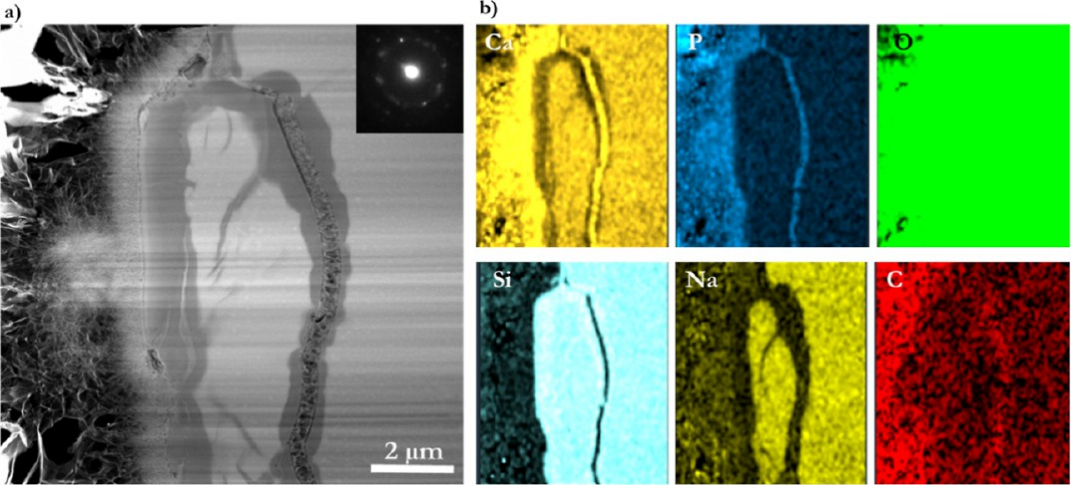
(a) TEM micrograph showing a cross-section of a BG-block reacted with modified AS for 1 h at 37 °C and the FFT of a high-resolution image (image inside). (b) Chemical elemental composition mapping was obtained from TEM–EDS measurements, where typical elements of interest were mapped.

**Figure 5:**
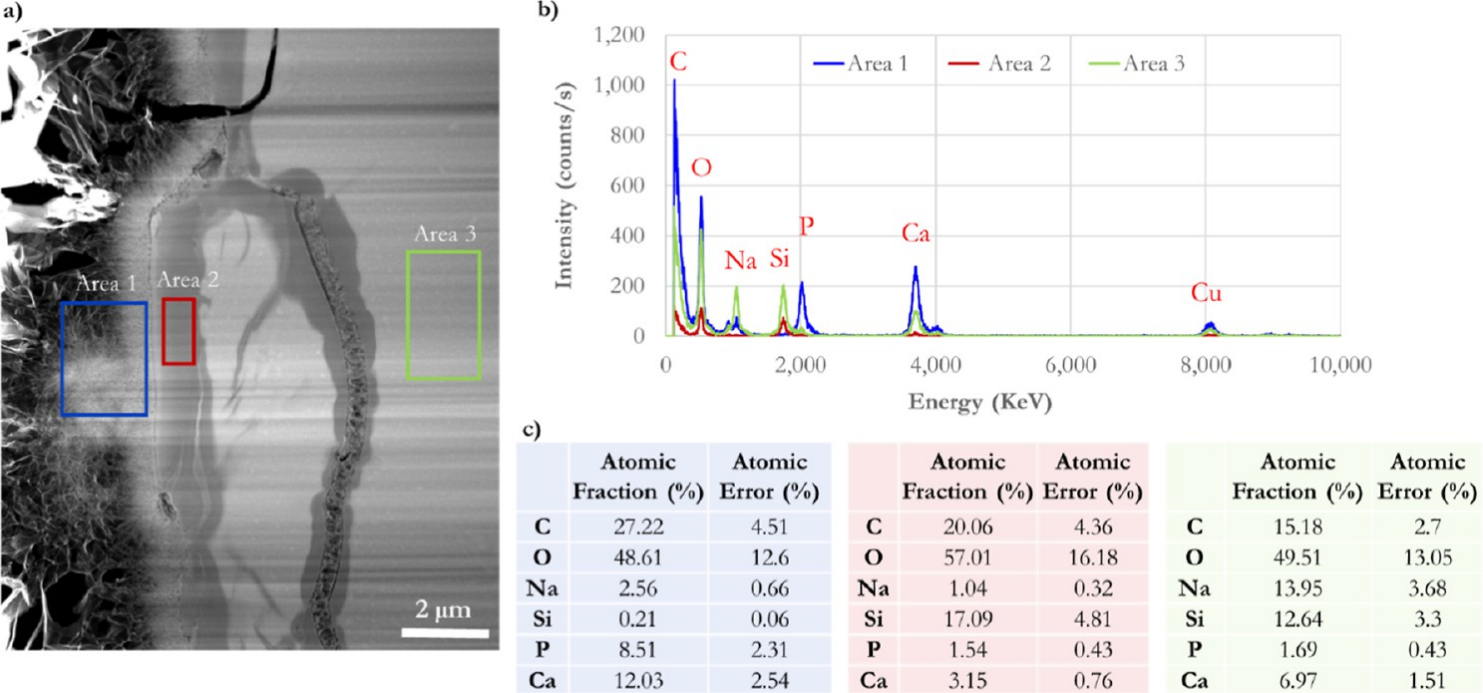
Post-TEM–EDS measurements of a cross-section BG-block reacted with modified AS for 1 h at 37 °C. (a) Three differentiated zones: CaP-formed material (squared blue area), a glass-depleted Si-rich layer (squared red area), and the nonreacted glass (squared green area). (b) Intensity spectra versus elemental energy of those distinctive zones formed during BG-dissolution as seen in the BG-block cross-section and highlighted in (a). (c) Different areas measured (highlighted in a) exhibit particular chemical elemental compositions corresponding to their fingerprint.

**Figure 6:**
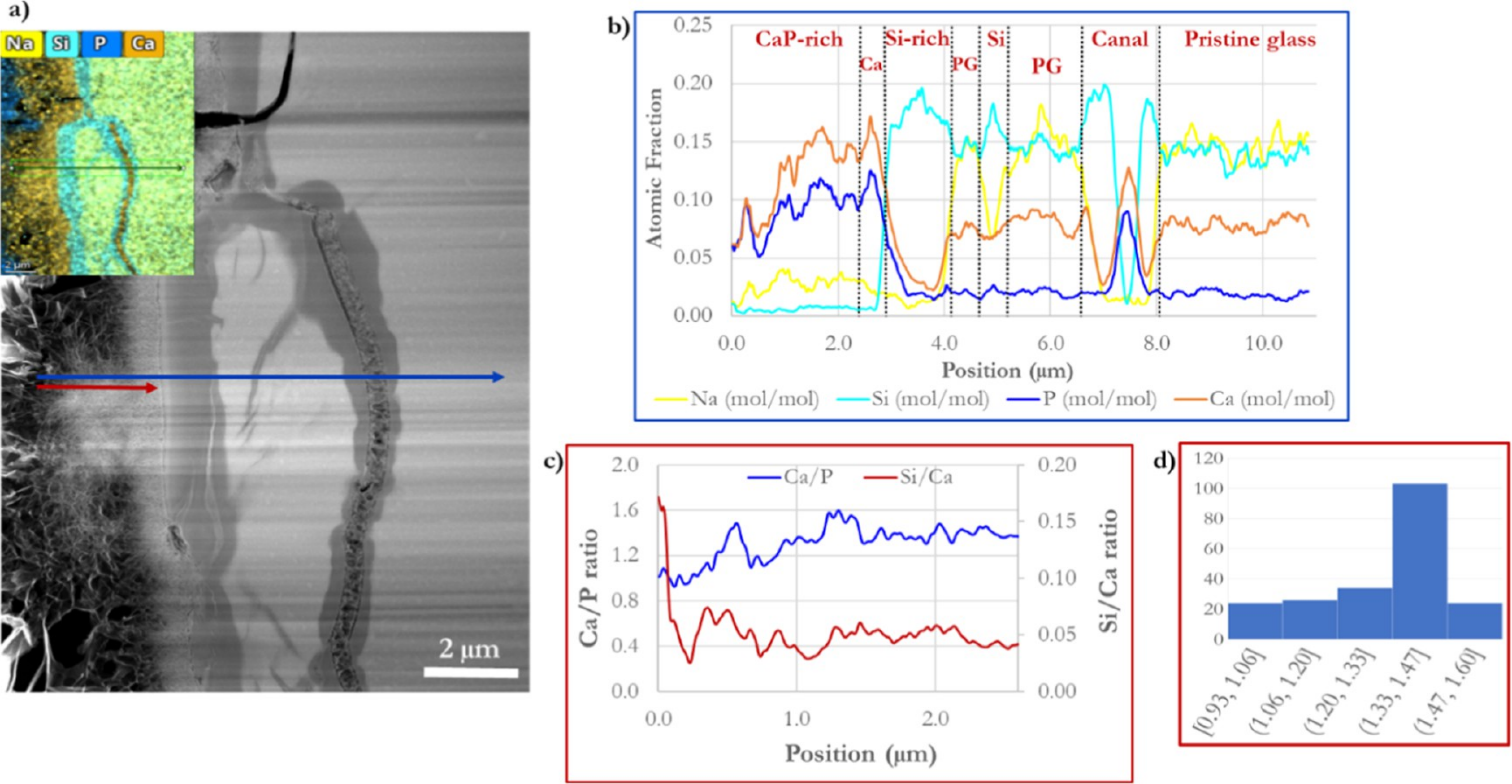
Post-TEM–EDS line scan profiles for different elements of interest of a cross-section BG-block reacted with modified AS for 1 h at 37 °C. (a) TEM micrograph showing the lines (blue and red) where the elemental atomic profiles were obtained. The inside image corresponds to EDS elemental mapping showing the different layers formed during BG-block dissolution in AS. (b) Graph corresponding to the blue line scan is divided according to the various zones obtained by EDS analysis. CaP-rich layer, Ca corresponds to the Ca^2+^ ions’ dense layer, Si-rich/Si corresponds to the Si-rich layer (altered glass), and PG corresponds to the pristine (unreacted) glass. (c) Graph corresponding to the red line scan shows Ca/P (primary axis) and Si/Ca (secondary axis) ratio profiles obtained by EDS analysis in the Ca-rich layer. (d) Ca/P ratio histogram obtained from the red line scan.

Figures 3–6 shows the results of the BG-block cross-section reacted with modified AS aqueous solution for 1 h at 37 °C and with an initial pH of ∼6.5.

The SEM micrographs in Figure 3a,b show the surface of the BG-block after immersion, which is fully covered with the CaP-formed material. Florets are visible on the surface (Figure 3a), and the formed material has a platelet-like structure. This morphology differs from the needle-like dense-formed material observed on ball-milled bioglass (BMBG) due to the intrinsic differences in the material type, one being a block and the other submicron particles; therefore, the ions’ diffusion rate during dissolution differs between materials.^60,65^

There are two kinds of CaP materials on the observed surface: CaP that has grown directly on the BG-block’s surface and growth CaP in the form of round globules originating perhaps from the CaP-supersaturated bulk solution that landed on the BG-block’s surface. Calcium phosphate formation occurs mostly heterogeneously, given a solid substrate, where the mineral precipitates and grows. However, a change in pH (from ∼6.5 to ∼9.6) caused by Na^+^ and Ca^2+^ ions released from BG triggers changes in the solubility of the CaP from the bulk solution.^60^ Therefore, homogeneous CaP nucleation in the bulk solution may also occur. However, heterogeneous nucleation is favored over homogeneous due to the reduced interfacial energy.^66^ The results show that Ca^2+^ ions from the glass exhibit dual behavior. Some may become trapped within the acidic silicate media on the BG surface when diffusing from the glass and reacting with PO_4_^3–^ ions from the bulk solution, forming CaP on the BG-block’s surface. Alternatively, some diffusing Ca^2+^ ions may move directly to aqueous solution. In addition, as AS is a CaP-containing aqueous solution in a supersaturated state, changes driven by BG dissolution, mainly pH, trigger amorphous calcium phosphate (ACP) homogeneous formation and precipitation as round blobs that land on the BG substrate, as shown in Figure 3a,b.

Aqueous solution infiltrates the pristine BG-block by about 1.2 μm h^–1^, showing CaP formation in the natural glass cracks. An altered glass layer (a dark gray area) and unreacted BG (a light gray area) are observed on the BG-block cross-sectioned (Figure 3c). Figure 3c shows that grown CaP presents diverse and distinctive morphologies.^26^ An inner layer has grown directly on the BG, displaying a spiky-packed nanocrystalline CaP structure. In contrast, the outer layer developed on the former has a looser platelet arrangement (floret-like), although a similar Ca/P ratio. Moreover, as indicated by the FFT of the high-resolution image (inside image Figure 3a), it corresponds to a crystalline material.

Figure 3c displays a natural BG-block crack/fissure (cyan-circled area). Due to capillarity, aqueous species freely move along the crack, creating an ion-depleted zone, a well-known phenomenon for this glass.^9^ The ion-depleted site along such a crack is characterized by a darker contrast that shows the aqueous-solution transport blueprint. The crack initially has open access through the BG-block’s surface. The closest part of the crack to the surface presents a more dense CaP formation. Then, it decreases depending on the aqueous species’ availability throughout the crack’s length. Remarkably, aqueous species penetration also creates the conditions for CaP formation observed along the fissure. On the other hand, the light-yellow circled area in Figure 3c reveals a thin layer, ∼50 nm wide, which corresponds to a dense Ca^2+^-ion layer, according to EDS analysis, may be reacted with PO_4_^3–^ likely resulting from a trapping effect of the acidic Si-rich surface layer.^31,67^

TEM–EDS measurements were performed on the cross-section of the BG-block reacted with AS. Maps of the chemical elemental composition were obtained (Figure 4). The figure distinctly illustrates the distribution of the ions, the differentiated zones created, and the ion diffusion patterns, where brighter element color is directly related to intensity. Ca^2+^ and PO_4_^3–^ ion maps are mainly concentrated on the CaP-rich layer formed on the BG-block’s surface, and the CaP developed along the internal glass crack/canal. Conversely, a high atomic fraction of Na^+^ ions remains in the unreacted glass layer (pristine glass); however, it is depleted in the Si-rich and CaP-rich layers although not entirely. The Si map shows what corresponds to the Si-rich layer and nonreacted glass. The C map shows carbon ions scattered; however, a higher atomic fraction is presented in the CaP-rich areas. The O map indicates that oxygen is pervasive as the layers formed are O containing. Moreover, the FFT image of the high-resolution image (image inside, Figure 4a) suggests that the developed material is crystalline, although its phase is not identifiable. The following figures show the atomic content of each layer in more detail.

Figure 5a highlights three differentiated zones of the cross-section of the BG-block reacted with an AS: CaP-formed material (squared blue area), a glass-depleted Si-rich layer (squared red zone), and the nonreacted glass (squared green area). Figure 5b displays the intensity spectra versus elemental energy of those distinctive zones formed during BG dissolution, as seen in the BG-block cross-section and highlighted in Figure 5a. The measured areas exhibit particular chemical elemental compositions and their atomic percentage (Figure 5c) corresponding to their fingerprint and are directly associated with the solution’s chemistry. It is worth noting that area 3 matches the atomic composition of unreacted BG, which has not been exposed to any dissolution. It is worth mentioning that electron beam-induced carbon contamination can explain the highly present carbon (C) in the three regions.^68^

The TEM-micrograph atomic contrast enables differentiation between the various surface layers obtained (Figure 6a), mirrored by the EDS line scans, as seen in Figure 6b. Two EDS line scans are shown in Figure 6a, underlining the BG-block-produced zones during dissolution (blue and red lines) to highlight the Ca/P ratio throughout the CaP-rich layer on the BG-block’s surface. The blue line EDS scan distinctly indicates the different elements of interest profiles throughout the different created surface layers during BG-block dissolution (Figure 6b). In comparison, the red line refers to Ca/P and Si/Ca ratios (Figure 6c), showing a patchy ratio of 1.4 ± 0.1 according to the histogram (Figure 6d).

BG dissolution is a complex multistage process in which mineral growth and dissolution are intrinsically interconnected.^19^ Notably, three significant zones can be differentiated in the BG-block cross-section: a CaP-rich zone formed on the BG’s surface, an altered glass zone^69^ and a nonreacted glass zone (pristine glass).

The CaP-rich zone is developed following solution-mediated dissolution, in which morphology and growth depend on the solution’s chemistry and temperature. The mineral growth presents a Ca/P ratio of about 1.4–1.5 (Figure 6c,d), indicating a calcium-deficient apatite-like material corresponding to a disordered apatitic material.^26^ The Ca/P ratio is consistent with the other published reports.^60,70^ The formed material contains considerable carbon absorbed or molecularly linked to the CaP-developed material. However, according to XPS measurements, such a carbon appearance mainly corresponds to CO_2_ absorbed from the environment; thus, the carbon molecularly bonded to the CaP formed layer is negligible, which is consistent with the free CO_2_ methodology utilized.^60^

During BG dissolution at a pH > 7, acidic silicate (silanol groups) may be formed in three ways: (i) water interaction/ion exchange with Na^+^ and Ca^2+^ ions resulting in Si–O–NBO protonation, which triggers pH increase;^14,71^ (ii) hydrolysis of the silica network (Si–O–Si) initiated by the basic pH occurring initially on the glass surface, which results in the silanol group formation or the release of silicic acid (H_4_SiO_4_) directly into the solution;^14,71^ (iii) hydrolysis of the glass which also triggers the formation of isolated low-molecular silica fragments that can be readily hydrolyzed and released into the solution. With a forming rate >1.4 μm h^–1^, the darker area next to the CaP-developed material corresponds to a heavy protonated site (a silica gel layer) with a shallow modifying-ion composition. This Si layer (altered zone) results in an acidic area with a high silanol group content,^31^ acting as favorable nucleating sides for CaP formation.^30^ This altered glass layer formation creates porous structures within the glass that hydrous species can easily penetrate,^11,13,31^ as shown in Figure 6. Lastly, a nonreacted zone corresponding to the pristine BG demonstrates an elemental composition similar to untouched BG, characterized by an almost 1:1 atomic fraction composition of Si/Na. The distance between any two analysis points is about 12 nm, giving a CaP-altered glass transition zone of about 200 nm. Figure 11 displays an example of this CaP–Si-rich layer (altered glass) interface formed during BG dissolution in more detail.

**Figure 7:**
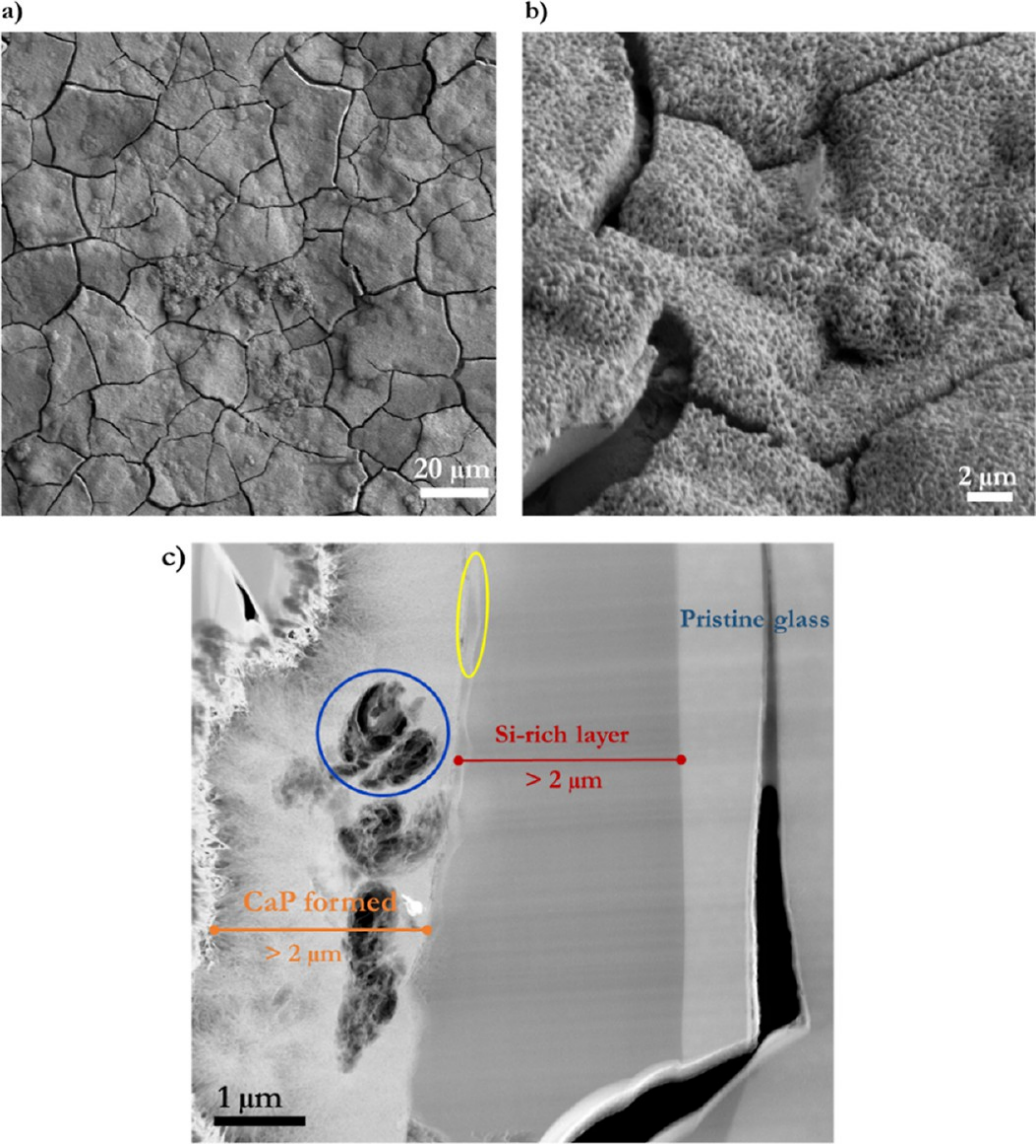
BG-block reacted with ammonium hydrogen phosphate (AmP) aqueous solution for 1 h at 37 °C. (a,b) SEM micrographs of the block’s surface after immersion. (c) BG-block cross-section shows some key characteristic results, such as the extent of CaP growth material (orange line) and the extension of the glass modifier ion depletion (red line). Two particular features are highlighted: the light-yellow circled area shows a thin layer, ∼100 nm wide, corresponding to a dense Ca^2+^ ion region; the blue-circled area corresponds to a morphologically odd CaP formation, which may be caused by rapid water migration during the crystallization transition process.

**Figure 8:**
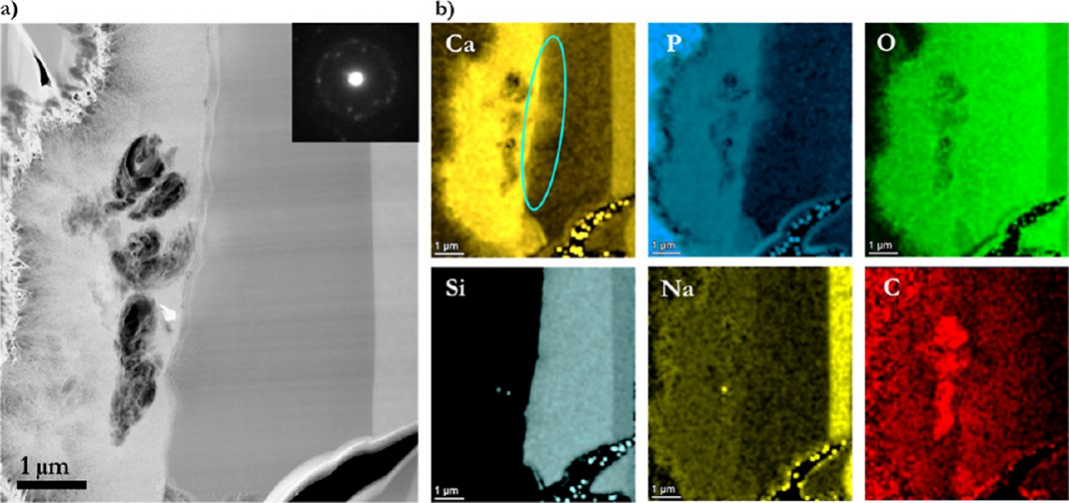
(a) TEM micrograph showing a cross-section of a BG-block reacted with AmP for 1 h at 37 °C and the FFT of a high-resolution image (image inside). (b) Chemical elemental composition mapping was obtained from TEM–EDS measurements, where typical elements of interest were mapped. The cyan-circled area on the Ca-map shows a denser CaP concentration than the rest of the CaP-rich zone.

**Figure 9:**
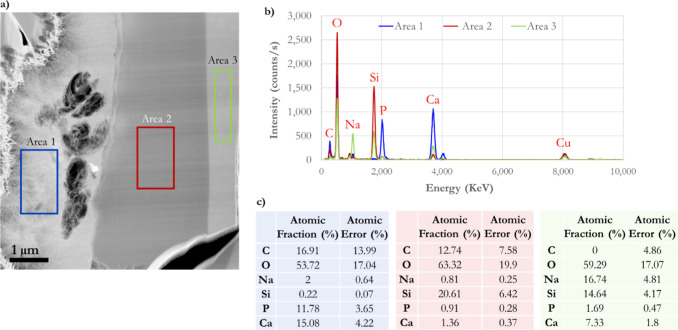
Presents post-TEM–EDS measurements of a cross-section BG-block reacted with AmP for 1 h at 37 °C. (a) Three differentiated zones: CaP-formed material (squared blue area), a glass-depleted Si-rich layer (squared red area) and the nonreacted glass (squared green area). (b) Intensity spectra versus elemental energy of the distinctive zones formed during BG dissolution as seen in the BG-Block cross-section and highlighted in (a). (c) Different EDS-TEM areas (highlighted in a) and particular chemical elemental compositions corresponding to their fingerprint.

**Figure 10:**
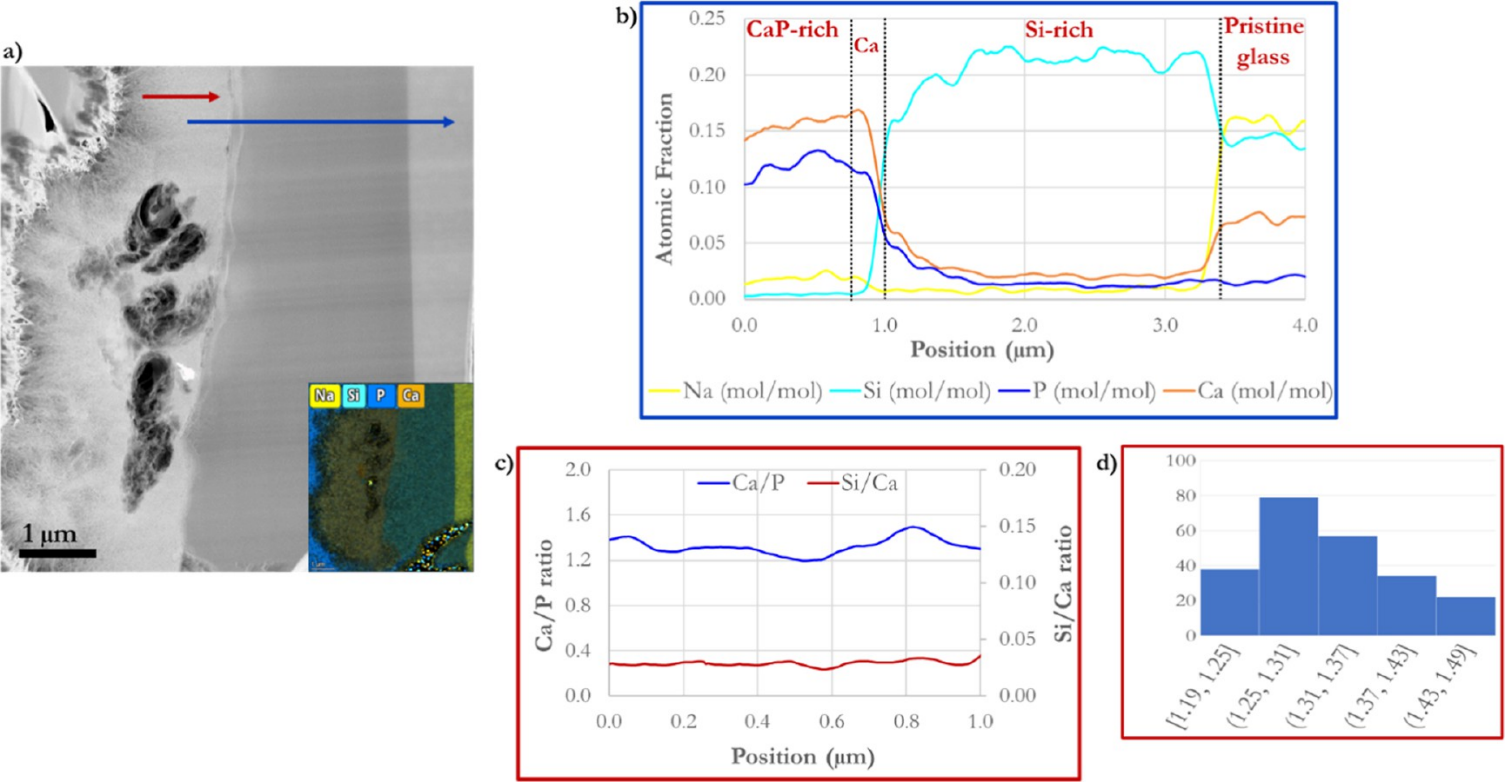
Post-TEM–EDS line scan profiles for different elements of interest of a cross-section BG-block reacted with an AmP solution for 1 h at 37 °C. (a) TEM micrograph showing the lines (blue and red) where the elemental atomic profiles were obtained. The inside image corresponds to EDS elemental mapping showing the different layers formed during BG-block dissolution in AmP—Ca, which refers to Ca^2+^ thin, denser layer. (b) Graph corresponding to the blue line scan is divided according to the various zones obtained by EDS analysis: Ca-rich, Si-rich (altered glass), and pristine (unreacted) glass. (c) Graph corresponding to the red line scan shows Ca/P (primary axis) and Si/Ca (secondary axis) ratio profiles obtained by EDS analysis in the Ca-rich layer. (d) Histogram of Ca/P ratio obtained from the TEM–EDS red line scan.

SEM micrographs in Figure 7a,b show the surface of the BG-block after immersion in AmP, which is fully covered with the CaP-formed material. However, the AmP solution does not contain Ca^2+^ ions; thus, all the Ca^2+^-ion consumption diffuses from the BG-block during dissolution conversely with the AS case. Two kinds of CaP material can be observed on the BG-block’s surface, as in the case of AS immersion (Figure 3a). The CaP material may have grown directly heterogeneously on the BG-block’s surface. Moreover, CaP may have formed homogeneously in the bulk solution as round globules that landed on the BG-block’s surface (Figure 7a,b). As in the previous case of a BG-block reacted with AS, calcium phosphate formation occurs mostly heterogeneously in a solid substrate (BG-block’s surface), where the mineral precipitates and grows. However, a change in pH (from ∼8 to ∼9) due to the Na^+^ and Ca^2+^ released during BG dissolution triggers changes in the solubility^72^ of CaP. Therefore, CaP formation may also occur homogeneously in the bulk solution, which may land on the BG-block’s surface, as illustrated in Figure 7a,b. However, due to the reduced interfacial energy, heterogeneous CaP formation is favored over homogeneous formation.^66^Figure 7c indicates that the grown CaP presents distinct and varied morphologies. An inner layer grown directly on the BG-block’s surface displays a spiky-packed structure.

In contrast, an outer layer that develops on the former has a looser, spiky arrangement but a similar Ca/P ratio. However, this CaP-formed material morphologically differs from the AS case (Figure 3c). The jam-packed CaP formation may allow hydrous molecules to become trapped within the CaP developed (dark-blue circled area Figure 7c), which may drain during dehydration/drying, remarkably leaving this porous growth material that morphologically differs from its surroundings.

The irregular cracks observed on the BG-block’s surface (Figure 7a) could be due to rapid dehydration when the reaction/dissolution process was forced to stop. These cracks were not directly observed on the BG-block’s surface reacted with AS (Figure 3a) as (i) the CaP platelet formation may disguise it and (ii) perhaps the morphology of the CaP formed in AS enables the hydrous molecules to leave without damaging the surface.

Figure 8 shows the chemical elemental composition maps obtained from BG-block cross-sectional TEM–EDS measurements after immersion in AmP aqueous solution. The figure distinctly exemplifies the distribution of the ions, the differentiated zones created, and the ion diffusion patterns. Ca^2+^ and PO_4_^3–^ ions mainly cluster on the CaP-rich layer formed on the BG-block’s surface. Conversely, although not wholly, Na^+^ ions are highly present in the pristine glass layer (unreacted glass) and depleted in the Si-rich and CaP-rich zones. The Si-map shows what corresponds to the Si-rich layer. The O-map displays pervasive oxygen as the different layers formed are O-containing. The C-map indicates carbon ions scattered; however, a higher atomic fraction is shown in the CaP-rich areas. Moreover, the FFT image of the high-resolution image (image inside Figure 6a) indicates that the formed material is crystalline, although the precise phase is not discernible. The following figures show many more details of the BG-block cross-sectioned.

Three differentiated zones are highlighted in Figure 9a: CaP-formed material (squared blue area), ion-depleted Si-rich layer (squared red area), and nonreacted glass (squared green area). Figure 9b displays the intensity versus elemental energy of the zones formed during BG dissolution, as seen in the BG-block cross-section and highlighted in Figure 9a. The measured areas exhibit particular chemical elemental compositions (Figure 7c) that correspond to their fingerprint and are directly associated with the solution’s chemistry. The atomic composition of the selected areas reflects the interaction of aqueous species within the glass, where aqueous-speciation mediation is crucial for ion mobility, species condensation, and further crystallization. Moreover, hydrous species play an essential role in the transition of ACP to apatite. The structural elimination of hydrous molecules from ACP forms part of this transition process in hydrated conditions to drive dehydration.^73−75^

TEM–EDS line scans (blue and red lines) show the element profiles across the different surface layers created during BG-block dissolution (Figure 10). An image scattering contrast identifies differentiated zones consistent with the EDS line scan display. As in AS and previously said, in the BG-block reacted with AmP solution, three zones can be differentiated within the cross-section: a calcium phosphate-formed zone on top of the BG-block, an altered glass area (ion depleted), and a non-BG reacted site (Figure 10a). The calcium phosphate material developed presents a growth rate of more than 2.2 μm·h^–1^.

The CaP-rich layer presents a Ca/P ratio of 1.3 ± 0.1 (Figure 10c,d), indicating a calcium-deficient material corresponding to an apatitic-like material.^26^ The material formed also contains a considerable amount of carbon absorbed or molecularly linked to the CaP-developed material. However, XPS analysis^60^ shows that this carbon mainly resulted from CO_2_ absorption during the BG-blocks’ exposure to the ambient air. Thus, carbon that may be molecularly linked to CaP is negligible. The relatively high level of carbon could be explained as cross-contamination when samples were exposed to air during manipulation/preparation.

The ion-depleted layer (altered glass), the darker area next to the CaP-developed material, presented a formation rate of more than 2 μm·h^–1^ (Figure 10b), corresponding to a heavily protonated site (a silica gel layer) crucial for CaP formation^30,31^ with a shallow modifying-ion composition. Lastly, the nonreacted zone corresponds to the pristine bioglass (Figure 10b), showing an elemental composition similar to nondissolved BG, characterized by an almost 1:1 atomic fraction composition of Si/Na.

**Figure 11:**
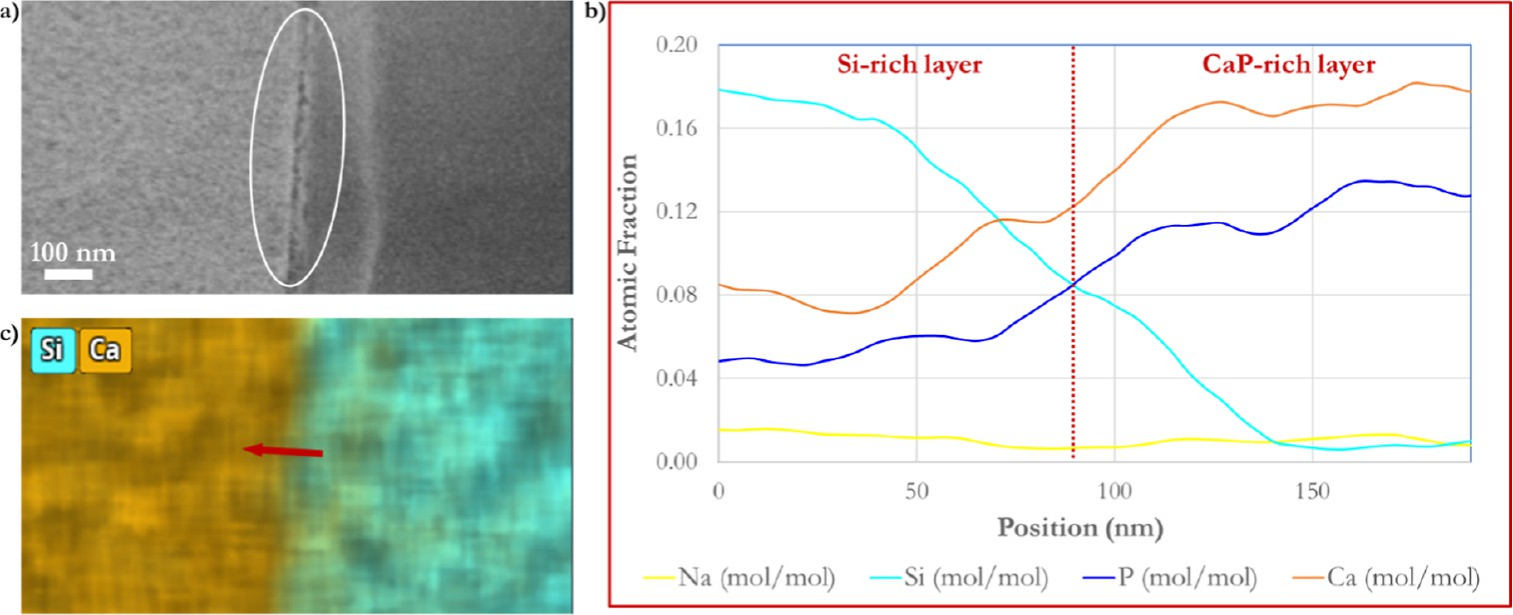
Thin Ca^2+^-rich layer of about 50 nm in the interface between CaP-formed material and the Si-rich layer observed during a BG-block cross-section reacted with an AmP solution for 1 h at 37 °C. (a) TEM micrograph, highlighting the Ca^2+^ dense, thin layer (white circled area). (b) TEM–EDS line scan profiles of elements of interest. (c) EDS elemental mapping showing the CaP-rich and Si-rich layers during BG-block dissolution in AmP and the interface formed. The location of the EDS line scan (red arrow) is also highlighted there.

A notable separating layer of about 50 nm in the interface between CaP-formed material and the Si-rich layer is observed (Figure 11). This thin layer may correspond to concentrated Ca^2+^ ions released from the glass that become trapped due to the acidic Si-rich layer produced during the dissolution process and may have already reacted with the PO_4_^3–^ ions from the liquid interface, as shown by EDS (Figure 10b). Moreover, a thin area between CaP and the ion-depleted layer can also be observed in the cross-section of the BG-block reacted with AS (Figure 6b). The thickness of the transition zone between the CaP-formed material and altered glass (Si-rich layer) is about 200 nm.

BG dissolution mechanisms usually involve cleaved silicate forming silicic acid (H_4_SiO_4_) that repolymerizes^15,76^ on the BG-block’s surface, forming silica gel that crucially interacts with Ca^2+^ and PO_4_^3–^ ions to form CaP. A repolymerized/reprecipitated silica gel may imply the formation of a differentiated new step-up layer with compositional characteristics that may stand out by electron imaging contrast or EDS. The results displayed in Figures 6 and 11 can be taken as examples of differentiated layers visible by contrast and highlighted by EDS. However, the results do not show a repolymerized/reprecipitated silica gel layer. If it exists, it would have to be very thin and just a few atomic layers (10–50) that serve as nucleating sides for CaP formation.^39,77^ On the other hand, a separation gap of about 15 ± 2 nm appears between the CaP-rich and Si-rich layers (Figure 11). This outcome may be due to mechanical stress, or perhaps as observed on BG particles (chapter 5^60^), to the inward dissolution of the Si-rich layer.

Nevertheless, a distinct Si signal gradient within such an interface (Figure 11) indicates that silicon remains within the transitional zone. The following equation shows that soluble silica can form poly metal silicates^78^ when linked to Ca^2+^ ions^11^
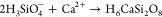


It has been identified that depending on the solution’s chemistry, Ca^2+^ and Si^4+^ ions interact and may form calcium silicates that homogeneously condense in the solution or become part of the silica gel layer formed during glass dissolution. Ca–Si interactions may increase the glass’s protection against water alteration/corrosion.^11,78−80^

In this study, using high-resolution TEM and EDS coupled with FIB cross-sectioning has enabled the research and analysis of the BG-block reacted with two phosphate-containing solutions. The produced cross sections exhibited a CaP growth rate of >2 μm h^–1^, where different zones were also formed during BG-blocks’ dissolution, and the CaP formed on the BG-block’s surface presented distinctive morphologies. However, these findings conflict with other works,^34,35,81^ where the CaP growth rate is different, no distinct zones are shown, and no distinct CaP morphologies were formed. These discrepancies may be due to differences in the imaging techniques or the solutions’ chemistry.

Although Na and Si ions released during BG dissolution are slightly higher in AS than in AmP solutions,^60,65^ the thickness of aqueous solution penetration (or depletion layer) in a BG-block reacted with AS solution in 1 h reaction, 1.2 ± 0.2 μm, is lower compared to AmP solution, 2 ± 0.2 μm. This outcome may be due to the slightly higher initial pH of the ammonium phosphate solution (pH ∼ 8) than that of the AS solution (pH ∼ 6.5).^82^ However, the exchange between divalent cations, already present in the AS solution, with glass network NBOs could be a bridging element between two NBOs, preventing further silica network hydrolysis.^11^

On the other hand, the thickness of CaP formed in a BG-block reacted with AS solution in a 1 h reaction, 4 ± 0.2 μm, is higher than that of AmP solution, 2.3 ± 0.2 μm. The already contained Ca^2+^ ions in the AS solution may have altered the kinetics of homogeneous and heterogeneous CaP formation and precipitation on the BG-block’s surface, accounting for the thicker layer of CaP material formed, as seen in Figure 3a and compared to Figure 7a. Furthermore, the difference in ionic strength and ion effect between solutions, where AS > AmP, are also crucial determining factors. In general, the observations obtained from this study strongly show the interconnection between interdiffusion, dissolution, and mineral growth during a BG dissolution/alteration process.^19,26^

In both cases of BG-blocks reacted with AS and AmP solutions, the rate of CaP formation on the BG-block’s surface was higher than the rate of the Si-rich layer formation, indicating that material growth may protect the glass against dissolution.^60,83^ In addition, it is also observed, although in low quantities, that Na and Si ions are constantly present within the CaP-formed material. Thus, the grown material may exhibit Na^70,84^ and Si^11^ substitutions that might be part of the formed CaP or another phase. These low quantities of Na^+^ and Si^4+^ on the developed material are consistent with results presented in other works.^60^

Based on the Hench model,^32^ many of the BG dissolution mechanisms described in the literature talk about cleaved silica in the form of silicic acid (H_4_SiO_4_) that repolymerizes/reprecipitates^76,85^ forming silica gel that crucially interacts with Ca^2+^ and PO_4_^3–^ ions to form CaP on the BG slab surface. This silica gel formation on the BG surface and the interdiffusion during BG dissolution resemble the alteration/corrosion process of other glasses.^39,63^ However, preliminary results of an experiment (section below: partially masked BG-block’s surface) show that CaP precipitation may be faster, preventing soluble silica condensation/reprecipitation on the top of the BG-block’s surface. Therefore, the formation of any noticeable condensed silica-gel layer stepping up. Nonetheless, the BG interdiffusion and dissolution processes create an acidic silicate-rich layer or perhaps a silica gel product of silicate network reorganization that promotes CaP formation and growth within the BG silica network.

In alternative configurations, such as BMBG dissolution, the significantly higher surface-area-to-volume ratio enhances ion diffusion and dissolution kinetics, as shown in our study of calcium phosphate mineralization driven by BMBG dissolution.^65^ This study also showed that BMBG dissolution strongly depends on the solution’s chemistry, where pH, ionic strength, and ion effect, are determining factors within the BG dissolution process. The increase in pH due to the release of BG’s modifying cations strongly favors CaP supersaturation, while the solubility of soluble silica increases, which is consistent with having an unnoticeable or perhaps a very tiny silica gel layer on the BG’s surface as observed in our results.

#### ToF-SIMS Measurements

**Figure 12:**
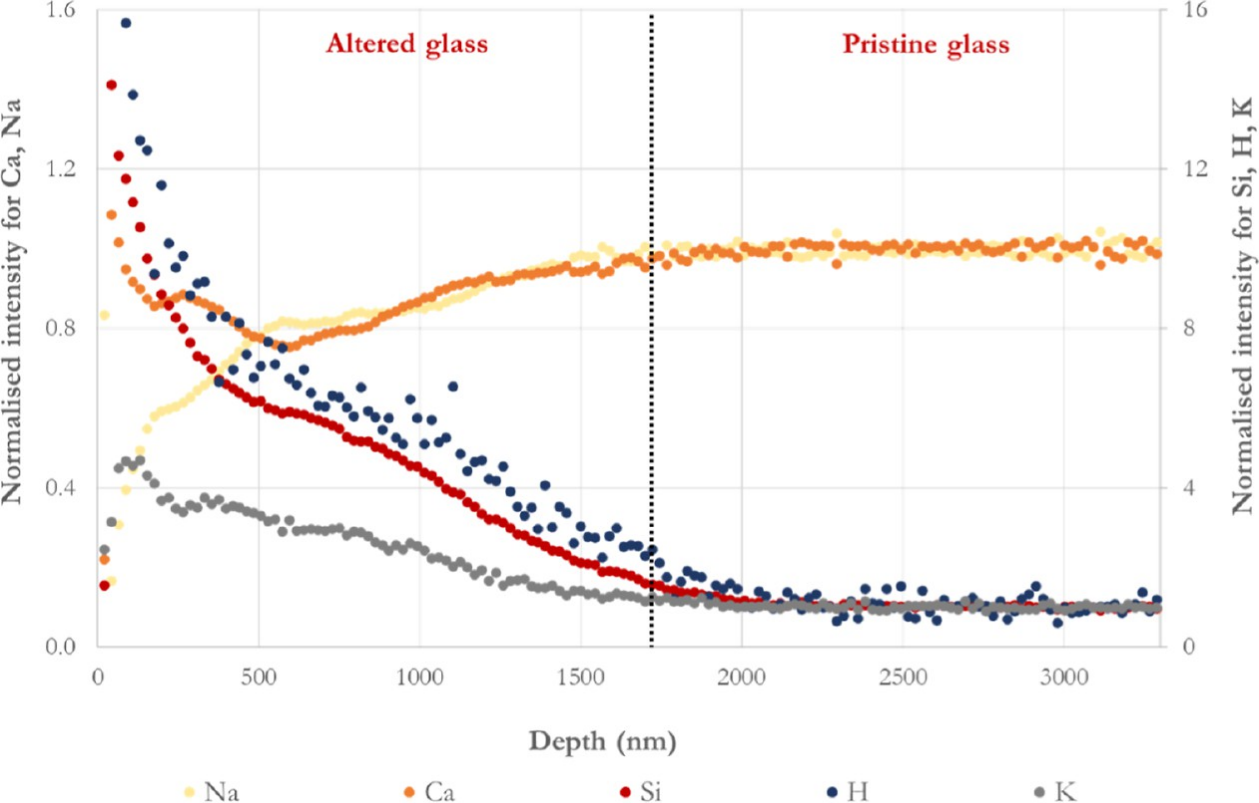
ToF-SIMS analysis, performed on the polished side of a BG-block after dissolution in silica-saturated solution at 37 °C and pH ∼ 7.3 ± 0.2. The interface between the alteration layer and the pristine glass is placed where Na^+^- and Ca^2+^-normalized concentration drop.

**Figure 13:**
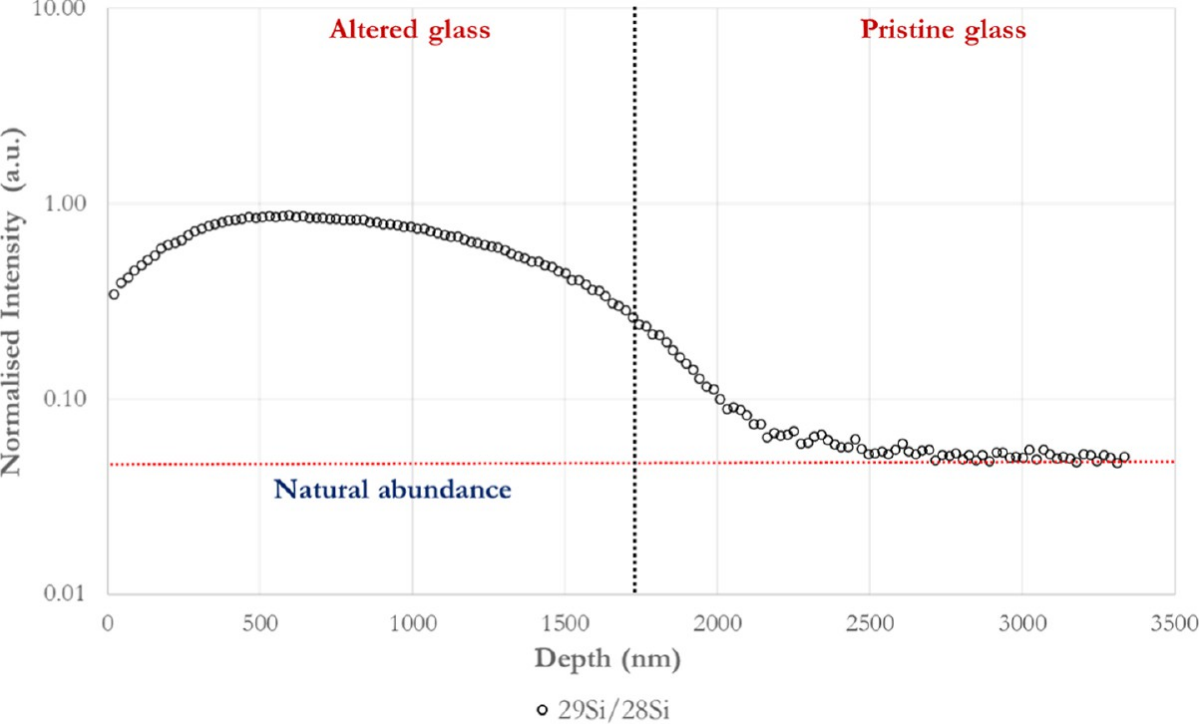
29Si/28Si profile of the ToF-SIMS analysis performed on the polished side of BG-block after dissolution in DI-water, silicon saturated at 37 °C and pH ∼ 7.3 ± 0.2.

Figure 12 and 13 exhibit ToF-SIMS measurements performed after reacting a BG-block in a SiO_2_-saturated solution tagged with isotopic ^29^Si at 37 °C for ∼5 h and at a pH of ∼7.3 ± 0.2. This analysis is considered qualitative due to the mobile character of all the species forming the BG matrix.

Figure 12 displays two distinctive zones: (i) an altered area, the product of an interdiffusion process due to the interaction/reaction of a BG-block’s surface with hydrous species, and (ii) an unreacted glass zone. 45S5 BG is considered a surface layer-forming material that is solution mediated. Thus, its dissolution is incongruent^16^ and mainly depends upon the intrinsic characteristics of the material, the solution’s chemistry, temperature, and pressure.^33,82,85,86^ However, when the BG’ surface area to solution volume ratio is substantially low (highly diluted), its dissolution is congruent.^60^

The mobile cations Ca^2+^ and Na^+^ present a similar profile between 600 and 1700 nm, indicating that they were likely leached out of the glass at a similar rate. Nonetheless, those profiles differ at the outer region (0–500 nm), suggesting that the outer layer corresponds to the precipitated zone (Figure 12), which is partly consistent with other observations.^60^ Both ions display a profile that decreases at a BG depth between ∼600 and 2000 nm, reading Figure 12 from right to left. The Ca^2+^ profile starts to increase from 0 to 600 nm, whereas the Na^+^ profile decreases with a sharper drop up to ∼600 nm when starting the sputtering process. Na^+^ ions are relatively depleted in the altered layer compared with the silicon profile. It is worth noting that the Na^+^ ions’ behavior is indirectly related to the H^+^ and Si^4+^ profiles, whereas Ca^2+^ cations behaved slightly differently, showing what resembles a form of accumulation near the BG surface.

Aqueous species, particularly H^+^ incursion into the glass, is rapid and seen along the altered area, gradually decreasing up to the unreacted BG area, which seems to mimic the Si^4+^ profile. In contrast, a sharp increase is observed from right to left (Figure 12) for H^+^ and Si^4+^ ions in the altered zone, presenting a similar outline. The contact of the material with a tagged isotopic ^29^Si-saturated solution indicates ^29^Si enrichment that exceeds its natural abundance. The ^29^Si/^28^Si profile increases within the altered layer to a depth of about 500 nm; after plateauing, it drops to reach its natural abundance within the unreacted BG area (3% error). This ^28^Si and ^29^Si exchange (Figure 13) signifies that ^28^Si from the silicate network is exchanged by ^29^Si to produce soluble silica (silicic acid) as a hydrous species.

Moreover, the ^29^Si/^28^Si profile confirms the formation of a silica-gel-rich layer that is highly protonated (Figure 13). Then (i) ^29^Si in the form of isotopic silicic acid (H_4_^29^SiO_4_) from the aqueous solution infiltrates the network through pores left by mobile cations that have been released to the solution^87^ and (ii) ^28^Si is replaced by a potentially condensed form of isotopic ^29^SiO_2_, probably silica gel. It has been shown that the second case is more likely to endure as the condensed isotopic species form new structures that become covalently bonded to the original network and, therefore, sorbed on the silicate pore walls.^39^ However, it has also been shown that such a sorbed isotopic ^29^Si, taken from the liquid phase, is easily exchangeable.^87^ Therefore, this Si-rich layer is driven by the dissolution of the BG and likely the reprecipitation of aqueous species.^14^ The zone between 1700 and 2500 nm could certainly correspond to a mixing zone due to the roughness of the outer and inner interfaces, instead of a real chemical gradient.

According to eq 1, a silicon tetrahedron, Q_3_/Q_2_, could be converted by condensation to a more stable Q_4_ by reacting with silicic acid (H_4_SiO_4_). As the solution is SiO_2_ saturated, this Si^4+^ exchange process could potentially modify the gel network’s connectivity (eqs 1 and 2) as the gel has a higher connectivity than the parent glass.^64^ However, further research is needed to determine this.
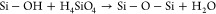
1
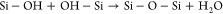
2

The isotopic silicon exchange indicates that an altered glass layer is formed, partly by freshly condensed silica from aqueous species. However, the amount of exchanged isotopic silicon and the precipitated amount of isotopic silica gel are unknown.

As the spiked isotopic Si-saturated aqueous solution contains potassium, it can be seen that this exogenous species, such as H^+^, also ingresses the altered glass and then steadily decreases along the altered zone. This K^+^ incursion into the altered silica network could be an exchange/replacement for Na^+^ and Ca^2+^ cations released from the silicate network during the BG dissolution process.

It is worth noting that water diffusivity into the glass strongly depends on the solution’s chemistry, where parameters, such as ionic strength, pH, and common ion effect profoundly influence the level of glass dissolution and, therefore, hydrous species ingress. On the other hand, solution conditions also distinctly steer the formation of CaP, steadily covering the glass surface during dissolution, potentially modifying the reactive surface of the pristine material underneath and the solid–liquid interaction (speciation of infiltrated water and liquid phase properties).^87^ One possible way to elucidate this is by doing further ToF-SIMS experiments, gradually varying the relevant parameters and conditions of the solution’s chemistry (pH, ionic strength, reactant concentrations, etc.).

According to results shown by Fourier-transform infrared spectroscopy (FTIR),^60^ and because BG dissolution is incongruent based on the CaP formation during dissolution, it is expected that after 5 h of BG dissolution in DI-W at 37 °C, ACP formation can be observed on the BG-block’s surface. However, the isotopic data do not show this. Neither does ToF-SIMS show whether soluble silica precipitates on the altered BG-block’s surface (Si-rich layer), forming a differentiated/new layer that may correspond to hydrated silica. Again, further ToF-SIMS experiments could help to elucidate this or offer a different approach.

#### Partially Masked BG-Block’s Surface

Due to the mobility of the Si^4+^ ions toward the aqueous solution in the form of H_4_SiO_2_ (soluble silica), during BG dissolution, it would be expected to (i) see a reduced height of the BG-block’s surface or (ii) the soluble silica to reprecipitate on the BG’s surface forming a step up. A particular approach was taken to elucidate whether during BG dissolution (i) a BG-block height would be reduced due to silica cleavage, in other words, a retreat of the initial interface can be measured or (ii) soluble silica from aqueous species would reprecipitate on a BG-block’s surface, forming a noticeable step. The results shown and obtained on the dissolution of submicron BG particles^65^ did not determine, whether soluble silica reprecipitates on the BG-block/particles’ surface. A dissolution experiment involving a partially masked BG-block surface was proposed and successfully implemented to address this ambiguity.

**Figure 14:**
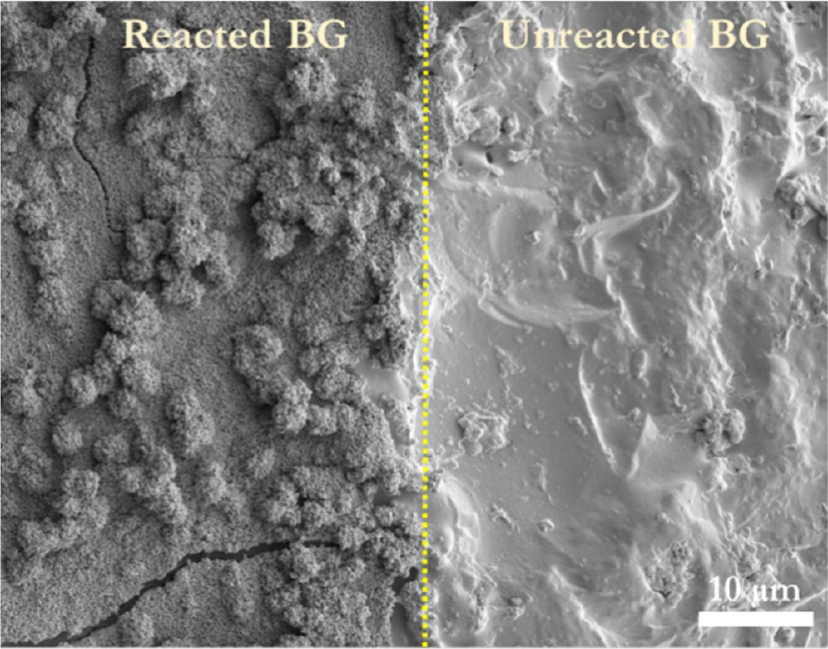
SEM micrograph showing the reacted and unreacted (glue-masked) sites of a BG-block after dissolution in a phosphate-containing solution at 37 °C for 1 h.

A BG-block’s surface was partially masked using an RTV silicon-based glue to make it hydrophobic.^88^ The BG-block was reacted with a phosphate-containing solution, (NH_4_)_2_HPO_4_, for 1 h at 37 °C. Figure 14 shows that the masked part of the block protected the covered area from reacting with the aqueous medium. Thus, the FIB cross-section that displays the interface between reacted and nonreacted BG was lifted and SEM-imaged. Moreover, a thinned and polished lamella was prepared and TEM-imaged.

**Figure 15:**
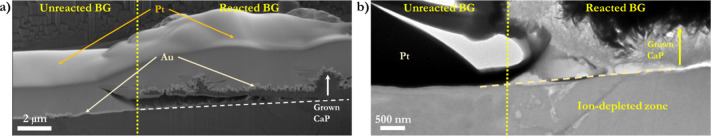
FIB-milled cross sections of the reacted and unreacted (glue-masked) sites of a BG-block after dissolution in a phosphate-containing solution at 37 °C for 1 h. (a) SEM image which corresponds to an unpolished FIB cross-section. (b) TEM image of a polished FIB cross-section. The arrows show the grown CaP material under these experimental conditions, and the polished lamella exhibits an ion-depleted zone on its unmasked side. The cross sections present gold (Au) and platinum (Pt) coatings to protect them from ion beam damage.

The SEM image (Figure 15a) shows the reacted and unreacted areas of the region of interest. However, this SEM image corresponds to a cross-section that is not yet polished; therefore, there is no differentiated contrast between the layers formed during dissolution, as shown in Figures 3 and 7. The reacted zone is fully covered with the CaP-developed material that presents a ribbon-like morphology, as appreciated from the top image, as shown in Figure 14. Two kinds of CaP-grown material cover the reacted BG-block’s surface: (i) a CaP that has grown directly on the BG surface and (ii) round CaP structures that seem to have landed on the BG-block’s surface from the aqueous species. FTIR and powder X-ray diffraction (PXRD) result show that the reacted area corresponds to a crystalline material that matches a nanocrystalline apatite material.^60,89,90^Figure 15 shows the difference in the CaP-grown material between the BG-reacted and the unreacted zones. The SEM micrograph shows no significant difference in the initial surface height between unreacted and reacted glass at this microscale; in other words, there is no retreat of the initial interface (Figure 15a). A higher-resolution image of the cross-section obtained from TEM (Figure 15b) again showed no difference between the reacted and unreacted surfaces, as no step-up is evident. However, this initial result is inconclusive, considering the rough and uneven surface of the BG-block (unpolished). The smooth and even platinum (Pt) and gold (Au) coatings on the unreacted side are worth noting. Conversely, the reacted site presents bumps along the Pt-coating, reflecting the CaP-round structures deposited on the BG-block’s surface and the Au-coating mirroring the morphological shape of the CaP-grown material.

Under experimental conditions, particularly concerning pH and temperature, the solubility difference between CaP and SiO_2_ is significant, favoring CaP supersaturation and, thus, spontaneous CaP precipitation on the BG-block’s surface from the aqueous species. Moreover, ACP rapidly precipitates (∼10 min) on the BG surface during dissolution in a phosphate-containing solution at 37 °C, as shown by FTIR and PXRD.^60,65^ Thus, ACP might be the material that first precipitates on the BG-block’s surface. If a SiO_2_ layer from the aqueous species precipitates on the reacted BG-block site, it might be a tiny layer of 10–50 atoms layers.^16^ As there is no retreat of the initial interface, this interface separates the Si gel from the CaP precipitate after alteration. Thus, the thickness of the Si layer gives a reasonable estimation of the thickness of the altered glass. A higher-resolution image given by TEM combined with EDS elemental analysis might help elucidate this.

## Summary

Solid-state analysis, including ToF-SIMS, FTIR, PXRD, etc., has shown significant insights into the mechanisms behind the BG structural and chemical changes during dissolution. Currently, two prevalent models describe glass dissolution: (i) a classical interdiffusion^91^ model and (ii) an interfacial dissolution/precipitation model.^37,87,92,93^ According to the techniques and analysis performed in this study, two distinct surface layers develop during BG dissolution: an outer CaP layer is formed by aqueous species precipitation on an inner Si-rich layer formed by diffusion and ion exchange. The ion-depleted inner layer, which occurs by the cation–proton exchange between the solid–liquid phases, is called the first stage of dissolution.^16,37^ The second stage corresponds to the hydrolysis/condensation reactions,^14,16^ where soluble silica may return to be part of the original network.

Additionally, due to the Ca^2+^-ion exchange, CaP precipitates on the Si-rich layer, forming the outer layer. The inner layer is directly linked to the external layer, as the acidic character of the Si-rich layer promotes the CaP outer surface-layer formation.^30,33^ The Si isotopic profile obtained from the ToF-SIMS results indicates that the Si-rich layer is likely formed by dissolution/reprecipitation. However, as established by the BG-block glue-masked experiment, there may be no soluble silica condensation/resorption on the edges/interface of the Si-rich layer/CaP layer. However, further investigations are needed to elucidate this. Moreover, the case of BG dissolution can be related to the classical dissolution model, where silica dissolution creates an ion-depleted zone and forms a silica gel by hydrolysis/condensation, promoting CaP formation on the BG’s surface.

## Conclusions

The slab BG work conducted in this study enabled quantitative measurements of the BG dissolution and its adhered phenomena. Cross sections of the BG-block reacted with two phosphate-containing solutions demonstrated that different zones were formed during BG-block dissolution, and the CaP formed on the BG-block’s surface presented distinctive morphologies depending on the aqueous solution used. Moreover, the length of CaP formed in a BG-block reacted with AS solution, 4 ± 0.2 μm h^–1^, is higher than that of AmP solution, 2.3 ± 0.2 μm h^–1^. In contrast, the length of aqueous solution penetration in a BG-block reacted with AS solution, 1.2 ± 0.2 μm h^–1^, is lower compared to AmP solution, 2 ± 0.2 μm h^–1^. Therefore, BG-blocks^60^ results may suggest that the grown CaP material on the BG-blocks’ surface may protect the glass against dissolution. In addition, it is also observed that Na^+^ and Si^4+^ ions were constantly pervasive within the CaP-formed material, although in low quantities. Thus, the growth material may exhibit Na^+^ and Si^4+^ substitutions that may be part of CaP or an additional phase. However, further research is required to fully resolve whether Na^+^ and Si^4+^ produce new phases or are molecularly linked to CaP.

According to the Hench^32^ model, the BG dissolution mechanism involves cleaved silica, forming silicic acid (H_4_SiO_4_—soluble silica) that repolymerizes/reprecipitates on the BG’s surface. Then, the silica gel formed crucially interacts with Ca^2+^ and PO_4_^3–^ ions to form CaP on the surface of the BG coupons (BG-blocks). This repolymerization/reprecipitation might imply relevant/noticeable changes on the BG’s surface, such as the likely formation of a differentiated new layer/step with compositional characteristics that would stand out by TEM/SEM contrast or EDS. The ToF-SIMS experiment indicated that during BG dissolution, an isotopic altered glass layer formed by the exchange between BG-silicon and spiked isotopic silicon from aqueous species. In this way, results suggest that during BG dissolution, (i) silica gel may be formed by in situ reorganization of the silicate network after the BG modifying ions are released, producing a tiny silica gel layer; (ii) if soluble silica from the aqueous species precipitates on the reacted BG-block site, it might be a tiny layer of 10–50 atoms layers. It has been shown that ACP rapidly precipitates (∼10 min) on the BG’s surface during dissolution in a phosphate-containing solution at 37 °C.^65^ Thus, ACP might be the material that first precipitates on the BG-block’s surface.

Although no BG-block surface changes (step-up/retreat) were observed due to soluble silica reprecipitation, the BG interdiffusion and dissolution processes create an acidic silicate-rich layer or perhaps a silica gel product of silicate network reorganization that promotes CaP formation and growth within the silica network.^60^

## Notes

This work was supported by the Engineering and Physical Sciences Research Council (EPSRC) [grant number 1803545]. Additional support was provided by Glaxo Smith Kline (GSK). The authors gratefully acknowledge their contributions to this research.
